# Machine Learning-Based Prediction of In-Hospital Complications in Elderly Patients Using GLIM-, SGA-, and ESPEN 2015-Diagnosed Malnutrition as a Factor

**DOI:** 10.3390/nu14153035

**Published:** 2022-07-24

**Authors:** Shan-Shan Ren, Ming-Wei Zhu, Kai-Wen Zhang, Bo-Wen Chen, Chun Yang, Rong Xiao, Peng-Gao Li

**Affiliations:** 1 Department of Clinical nutrition, Beijing Hospital, National Center of Gerontology, Institute of Geriatric Medicine, Chinese Academy of Medical Sciences, Beijing 100730, China; renshanshan4288@bjhmoh.cn (S.-S.R.); zhumwei@bjhmoh.cn (M.-W.Z.); 2 The Key Laboratory of Geriatrics, National Center of Gerontology, National Health Commission, Beijing Hospital, Beijing Institute of Geriatrics, Institute of Geriatric Medicine, Chinese Academy of Medical Sciences, Beijing 100730, China; 3 School of Public Health, Capital Medical University, Beijing 100069, China; zhangkaiwen@ccmu.edu.cn (K.-W.Z.); chenbowen@ccmu.edu.cn (B.-W.C.); yangchun@ccmu.edu.cn (C.Y.); xiaor22@ccmu.edu.cn (R.X.); 4 Beijing Key Laboratory of Environmental Toxicology, Beijing 100069, China; 5 Beijing Key Laboratory of Clinical Epidemiology, Beijing 100069, China; 6 Sir Run Run Shaw Hospital, Hangzhou 310000, China

**Keywords:** GLIM, SGA, ESPEN 2015, malnutrition, in-hospital complications, elderly patients

## Abstract

Background: Malnutrition is prevalent in elderly inpatients and is associated with various adverse outcomes during their hospital stay, but the diagnosis of malnutrition still lacks widely applicable criteria. This study aimed to investigate the association of malnutrition diagnosed with the SGA, ESPEN 2015, and GLIM criteria, respectively, with in-hospital complications in elderly patients. Method: Hospitalized patients over 65 years old who had been assessed with the SGA guideline for malnutrition at admission were retrospectively recruited from a large observational cohort study conducted in 34 level-A tertiary hospitals in 18 cities in China from June to September 2014. Malnutrition was then retrospectively diagnosed using the GLIM and ESPEN 2015 criteria, respectively, for comparison with the results of the SGA scale. The risk factors for malnutrition were analyzed using logistic regression, and the value of the three diagnostic criteria in predicting the in-hospital complications was subsequently explored using multivariate regression and the random forest machine learning algorithm. Results: A total of 2526 subjects who met the inclusion and exclusion criteria of the study were selected from the 7122 patients in the dataset, with an average age of 74.63 ± 7.12 years, 59.2% male, and 94.2% married. According to the GLIM, SGA, and ESPEN 2015 criteria, the detection rates of malnutrition were 37.8% (956 subjects), 32.8% (829 subjects), and 17.0% (429 subjects), respectively. The diagnostic consistency between the GLIM and the SGA criteria is better than that between the ESPEN 2015 and the SGA criteria (Kappa statistics, 0.890 vs. 0.590). Logistic regression showed that the risk of developing complications in the GLIM-defined malnutrition patients is 2.414 times higher than that of normal patients, higher than those of the ESPEN 2015 and SGA criteria (1.786 and 1.745 times, respectively). The random forest classifications show that the GLIM criteria have a higher ability to predict complications in these elderly patients than the SGA and ESPEN 2015 criteria with a mean decrease in accuracy of 12.929, 10.251, and 5.819, respectively, and a mean decrease in Gini of 2.055, 1.817, and 1.614, respectively. Conclusion: The prevalence of malnutrition diagnosed with the GLIM criteria is higher than that of the SGA and the ESPEN 2015 criteria. The GLIM criteria are better than the SGA and the ESPEN 2015 criteria for predicting in-hospital complications in elderly patients.

## 1. Introduction

Malnutrition is commonly seen in elderly patients hospitalized for various acute and chronic diseases [1]. It has many causes, such as decreased food intake of the patients and adverse impacts of diseases [2]. It is known that many diseases are accompanied by a systemic inflammatory and catabolism-prone status that accelerates the development of malnutrition [3]. In turn, malnutrition can inflict a variety of complications such as infection, anemia, and anastomotic fistula, resulting in prolonged hospital stays, increased mortality, and increased medical expenses, forming a malicious cycle [1,4,5]. Therefore, preventing the occurrence of malnutrition is vital for breaking this cycle and improving the clinical outcomes of patients.

However, to date, widely applicable diagnostic criteria for malnutrition that can be used in a wide range of clinical settings have not been proposed. Several diagnostic criteria are now used in clinical practices around the world, such as the subjective comprehensive assessment (SGA) scale [6,7], the framework established by the European society for clinical nutrition and metabolism in 2015 (ESPEN 2015) [8], and the global leaders’ initiative on malnutrition (GLIM) criteria [9]. 

The SGA scale is a comprehensive method for malnutrition diagnosis. It incorporates both subjective and objective evaluations of the patients based on their medical history, physical examination, nutritional status, changes in body weight and dietary intakes, gastrointestinal symptoms, physical activity, diseases, and measurement of the skinfold thickness of the triceps and the existence/inexistence of edema [6,7]. SGA has shown excellent accuracy in predicting prolonged hospital stay, re-hospitalization, and mortality in various patients [10,11]. However, the criteria are relatively challenging for clinicians to implement because they need to be trained before they can do it proficiently. Because the accuracy of the evaluation is affected by the professional level of the evaluator, the SGA scale is not presently widely adopted in many clinical settings by clinicians [12].

To standardize and simplify the diagnosis of malnutrition, the ESPEN 2015 framework was proposed. It is simple to use because it consists of only objective criteria such as low body mass index (BMI), unconscious weight loss, and reduction in the fat-free body mass index (FFMI) [8]. As the first attempt to reach a global consensus on malnutrition diagnosis, the criteria only cover the phenotypic features of malnutrition and have omitted the etiology of it, further optimization is needed before they can be widely accepted [2].

The GLIM guideline was formally proposed by several major clinical nutrition societies in 2018 to reach a global consensus on malnutrition diagnosis [9]. Compared to ESPEN 2015, GLIM covers not only the phenotypic factors of malnutrition but also its etiology. GLIM consists of three phenotypic criteria (body mass index, weight loss, and muscle mass loss) and two etiology criteria (decreased food intake/impaired nutrient absorption and inflammation). As long as at least one phenotypic criterion and one etiological criterion are met, malnutrition can be diagnosed. Since its publication, GLIM has been assessed by many researchers for its accuracy in predicting clinical outcomes in patients with cancer, cardiovascular diseases, surgery, and other diseases in comparison with other methods [13,14,15,16]. It has been found that the incidence of malnutrition diagnosed by GLIM is higher than SGA and ESPEN 2015 [17,18]. Its accuracy in predicting postoperative complications and total mortality in patients with surgical diseases is also higher than the latter two [18]. However, its accuracy in predicting some specific clinical outcomes such as the risks of falls and fractures and hospital admission in community elderly is sometimes inferior to SGA and ESPEN 2015 [19,20]. It means that more clinical validation studies are needed to establish its superiority in predicting specific clinical outcomes in specific populations.

Moreover, because the diagnostic results of the criteria mentioned above often differ, it is often confusing for clinicians when they want to select a method to assess the nutritional status of patients and predict their clinical outcomes and accordingly optimize their treatment regimens [1,2,21]. To address the problem, it is necessary to test the value of these diagnostic criteria in different clinical scenarios in a case-by-case manner before a consensus is reached on a widely accepted, universally applicable diagnostic criterion for malnutrition.

This study aimed to test the prevalence of malnutrition in elderly patients admitted to hospitals for tumors, digestive system diseases, nervous system diseases, etc., and to compare the value of the three diagnostic criteria for malnutrition in predicting in-hospital complications. We retrospectively analyzed the data of 7122 elderly inpatients aged over 65 years at admission. They were initially recruited for a large cohort study that took place in 34 level-A tertiary hospitals in China. After data screening, 2526 patients who had completed the nutritional assessment with the SGA scale at admission and met the inclusion/exclusion criteria of the current study were included in the final evaluation and were diagnosed retrospectively with the ESPEN 2015 and GLIM criteria, respectively. Our results indicate that, compared with the SGA and ESPEN 2015 criteria, more patients were diagnosed with malnutrition with the GLIM criteria. The accuracy of the GLIM criteria in predicting the in-hospital complications of these patients was also superior to the SGA and the ESPEN 2015 criteria.

## 2. Materials and Methods

### 2.1. Population

The data of this study came from a large-scale prospective observational cohort study conducted in 34 level-A tertiary hospitals in 18 cities in China from June to September 2014 with 7122 subjects [7,22]. According to the hospital grading system in China, there are three tiers of hospitals. Each tier is further subdivided into three subsidiary levels, A, B, and C, based on hospital service, size, management, quality, safety, facility, medical technology, etc. To date, tertiary A is the highest level that most general hospitals can obtain. The admission and follow-up data of these subjects were screened according to the inclusion and exclusion criteria of the current study. The inclusion criteria are patients who were 65 years old or above in the internal medical or surgical wards with self-consciousness who have signed written informed consent. The exclusion criteria were patients who were in the wards for <7 days or >30 days, and patients without anthropometric results. All subjects gave their informed consent before they participated in the study. The study was conducted in accordance with the Declaration of Helsinki, and the protocol was approved by the ethics committee of the Beijing Hospital (No. 2014BJYYEC-022-02) and registered in the China Clinical Trial Registration Center (Registration No. ChiCTR-EPC14005253).

### 2.2. Data Collection

A standardized research protocol was adopted in the above-mentioned cohort study and the present study. Within 24 h of admission, patients were assessed by trained clinicians with the nutritional risk screening-2002 (NRS-2002) scale [23] and then diagnosed for malnutrition with the subjective global assessment (SGA) guideline [6,7]. Malnutrition as defined by the ESPEN 2015 guideline [8] and the GLIM framework [9] was retrospectively determined by reviewing the subjects’ data. The data collected in this study include (1) demographic parameters: gender, age, marital status, and educational level; (2) Reasons for hospitalization, medical history, weight loss, and food intake; (3) Anthropometric parameters: height, weight, mid-upper arm circumference, calf circumference, and grip strength as measured by standard methods; (4) Laboratory parameters: whole blood cell counts and blood biochemistry including albumin, total protein, pre-albumin, triglyceride, cholesterol, etc. The primary outcome of this study was the occurrence of various complications within 30 days of admission. Complications are defined as any deviation from the ideal treatment process during hospitalization such as infection, anastomotic leakage, anemia, electrolyte disorder, myocardial infarction, etc., but not including the untreated primary diseases. Infectious complications are defined as the presence of pathogens in sterile tissues that were confirmed by culture, or the presence of clinical symptoms and signs, and radiological or hematological evidence of infection. The secondary outcomes of the current study include elongation of hospital stay, being transferred to intensive care unit (ICU), days spent in ICU, total hospitalization expenses, and death during hospitalization.

### 2.3. Diagnostic Criteria for Malnutrition

#### 2.3.1. The SGA Criteria

Malnutrition was diagnosed for patients using the SGA guideline within 24 h of hospital admission by a trained physician in terms of the following eight criteria: changes in body weight in the past two weeks; changes in food intake in the past two weeks; gastrointestinal symptoms lasting for over two weeks; physical activity; diseases and their relationships with the nutritional needs; reduction of subcutaneous fat; muscle atrophy; and ankle edema. Each result is divided into three levels (A, B, and C) in terms of severity. If at least 5 out of the 8 items are rated as B or C these patients can be rated as moderate or severe malnutrition, respectively, the sum of which is deemed the total cases of malnutrition diagnosed by the SGA criteria in the current study.

#### 2.3.2. The ESPEN 2015 Criteria

There are two steps. ① Screening: subjects with an NRS2002 score ≥ 3 points are considered to have nutritional risks and they need to be further diagnosed with the criteria in the second step; ② Diagnosis: For those with a nutritional risk, they can be diagnosed with malnutrition according to any one of the following criteria. (1) Body mass index < 18.5 kg/m^2^. (2) An unintentional weight loss combined with an age-related body mass index < 20 kg/m^2^ for those < 70 years of age, or < 22 kg/m^2^ for those ≥ 70 years of age.

#### 2.3.3. The GLIM Criteria

There are two steps. ① Screening: subjects with an NRS2002 score ≥ 3 points are considered to have nutritional risks and they need to be further diagnosed with the criteria in the second step; ② Diagnosis: malnutrition can be diagnosed if the subject meets at least one of the following phenotypic criteria and one etiological criterion. Phenotypic criteria: (1) Weight loss: unconscious weight loss of more than 5% in the past 6 months, or loss of more than 10% of the body weight in the past 6 months. (2) BMI reduction: the thresholds for Asians in the GLIM framework are a BMI < 18.5 kg/m^2^ for people < 70 years old and a BMI < 20 kg/m^2^ for people ≥ 70 years old. (3) Muscle mass reduction: because there are no data on the body composition of the subjects in this cohort, reductions in calf circumference and grip strength are used as alternative indicators to evaluate the reduction of muscle mass [24]. The calf circumference of men < 34 cm and women < 33 cm were taken as the threshold of calf circumference reduction, and the grip strength of men lower than 28 kg and women lower than 18 kg were used as the threshold of grip strength reduction according to the consensus of the Asian working group for sarcopenia (AWGS) 2019 [25]. Etiological criteria: (1) Reduced food intake or presence of digestive and absorption disorders: energy intake is reduced by more than 50% for more than one week, or energy intake is reduced for more than two weeks, or accompanied by chronic gastrointestinal diseases that affect food intake and/or absorption; (2) Disease burden/inflammation: there are acute and chronic inflammation-related diseases or injuries, which are evaluated according to the subject’s history of acute and chronic diseases at admission. In this study, a decreased serum albumin level (<35 g/L) was used as an indicator of inflammation [8,26]. Severity rating: those who meet one of the following criteria can be diagnosed with severe malnutrition: (1) weight loss > 10% in the past 6 months, or weight loss > 20% in the past 6 months or more; (2) a BMI < 17.8 kg/m^2^ for those < 70 years of age, or a BMI < 17.0 kg/m^2^ for those ≥ 70 years of age. Those who do not meet the criteria of severe malnutrition can be diagnosed with moderate malnutrition. Moderate malnutrition and severe malnutrition were combined to calculate the total number of malnutrition cases diagnosed by the GLIM framework in the current study.

### 2.4. Statistical Analysis

The IBM SPSS statistical software, version 26.0, was used to analyze the data. The normality of variables was evaluated by the Shapiro–Wilk test. Continuous variables conforming to the normal distribution are expressed as mean ± standard deviation (SD), and the means between two groups are compared by the student’s t-test. Continuous variables that do not conform to the normal distribution are expressed as the median and the quartile deviation (QD), and the Mann–Whitney U test is used to compare blood cell counts, ICU stays, and hospitalization expenses between groups. Categorical variables were expressed as frequencies and percentages, and compared by the Chi-square test. Logistic regression was used to assess the risk factors of malnutrition. The contribution of each exposure variable to the outcome variable was evaluated according to its odds ratio (OR). The relationship among the three diagnostic criteria was evaluated in terms of accuracy, sensitivity, and specificity. The consistency between the two criteria was evaluated according to the Kappa statistics. A Kappa score between 0 and 0.20 indicates weak consistency; 0.20–0.4 indicates low consistency; 0.4–0.6 indicates medium consistency; 0.6–0.8 indicates good consistency; 0.8–1 indicates excellent consistency. The association of the malnutrition diagnosed by the three diagnostic criteria with the incidences of different in-hospital complications was evaluated according to the OR obtained from the logistic regression. Spearman’s rank correlation was used to analyze covariates of the exposure and outcome variables. All statistical tests were bilateral. A *p*-value < 0.05 was considered statistically significant.

### 2.5. Machine Learning Analysis

The R software, version 3.6.3, was used to calculate the mean decrease in accuracy and the mean decrease in Gini coefficients for the three diagnostic criteria using the random forest algorithm. To generate the machine learning models, the dataset was split into 75% for training and 25% for testing. The model training process was repeated 500 times and the average performance metrics were determined. Each training run produces an individual decision tree. All decision trees form a random forest. Mean decrease in accuracy is the degree of decrease in the accuracy of random forest prediction after changing the value of a variable into a random number. Mean decrease Gini is to calculate the influence of each variable on the heterogeneity of the observed values at each node of the decision tree, to compare the importance of the variables. The higher value indicates the higher relative importance of the variable in the model.

## 3. Results

### 3.1. General Characteristics of the Subjects

**Figure 1:**
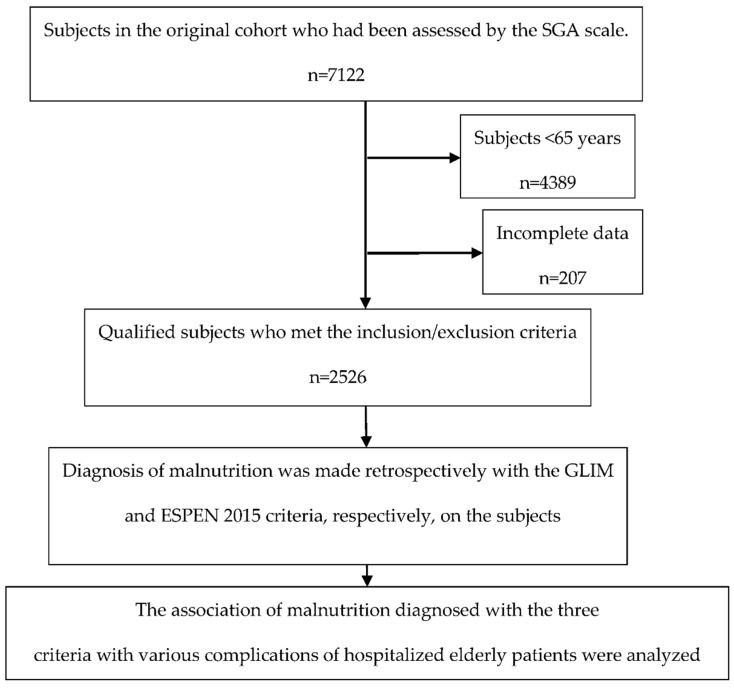
Flow chart of the study.

After examining the registration data of the 7122 hospitalized patients enrolled in the previous cohort study, 4389 patients who were < 65 years of age at enrollment and 207 patients who lack the data on the primary and secondary outcomes of the present study were excluded. As a result, 2526 patients were finally enrolled in the current study (Figure 1). The average age of the subjects was 74.63 ± 7.12 years. 59.2% of the subjects were male and 94.2% were married. The main reasons for hospitalization include tumors (37.5%), digestive system diseases (18.3%), and nervous system diseases (17.0%).

### 3.2. Prevalence of Malnutrition

The prevalence of malnutrition diagnosed with the GLIM framework was higher than that of the SGA scale and the ESPEN 2015 guideline. According to the GLIM criteria, 956 subjects (37.9%) were diagnosed with malnutrition, of which 600 (23.8%) were moderately malnourished and 356 (14.1%) were severely malnourished. According to the SGA criteria, 829 subjects (32.8%) were diagnosed with malnutrition, of which 706 (28.0%) were moderately malnourished and 123 (4.9%) were severely malnourished. According to the ESPEN 2015 criteria, 429 subjects (17.0%) were diagnosed with malnutrition.

### 3.3. Differences between Malnourished and Normal Subjects

Table 1 shows that the age and the proportion of married subjects from the malnourished subjects are higher than those of non-malnourished subjects (*p* < 0.05) no matter which diagnostic criteria were used. There was no significant difference between the two groups (*p* > 0.05) in gender and educational level. In terms of the anthropometric parameters, the body weight, BMI, grip strength, mid-upper arm circumference, and calf circumference of malnourished people are lower than those of non-malnourished people (*p* < 0.05) no matter which diagnostic criteria were used. As to the hematological parameters, the whole blood lymphocyte count and the serum concentrations of hemoglobin, total protein, albumin, pre-albumin, triglyceride, and total cholesterol in malnourished patients are lower than those in non-malnourished patients (*p* < 0.05) no matter which diagnostic criteria were used. Concerning the hospitalization reasons, the proportions of the malnourished patients who were hospitalized due to tumors and respiratory diseases are higher than that of non-malnourished patients (*p* < 0.05), while the proportions of the malnourished patients who were admitted due to nervous system diseases and bone and joint diseases are lower than that of non-malnourished patients (*p* < 0.05) no matter which diagnostic criteria were used.

### 3.4. Diagnostic Consistency between the Criteria

**Figure 2:**
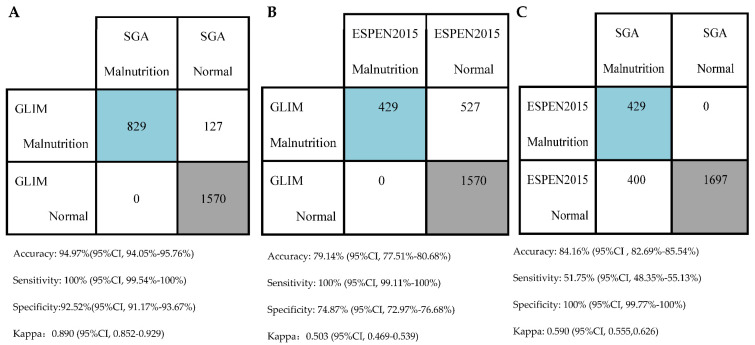
Diagnostic consistency between the criteria. (**A**) Consistency between GLIM and SGA. (**B**) Consistency between GLIM and ESPEN 2015. (**C**) Consistency between ESPEN 2015 and SGA. GLIM, Global Leadership Initiative on Malnutrition; SGA, subjective global assessment; ESPEN 2015, the 2015 consensus statement by the European Society for Clinical Nutrition and Metabolism.

Figure 2 shows the diagnostic consistency of the three criteria. Taking SGA as the gold standard, the accuracy of GLIM in diagnosing malnutrition is 94.97% (94.05%, 95.76%); the sensitivity is 100% (99.54%,100%); the specificity is 92.52% (91.17%, 93.67%). The Kappa consistency statistic is 0.890(0.852, 0.929) (Figure 2A), showing that GLIM is excellently consistent with SGA. Compared with ESPEN 2015, the accuracy of GLIM in diagnosing malnutrition is 79.14% (77.51%, 80.68%); the sensitivity is 100% (99.11%, 100%); the specificity is 74.87% (72.97%, 76.68%). The Kappa coefficient is 0.503 (0.469, 0.539) (Figure 2B), indicating that the diagnosis results of ESPEN 2015 and GLIM are moderately consistent with each other. Taking SGA as the gold standard, the accuracy of ESPEN 2015 in diagnosing malnutrition is 84.16% (82.69%, 85.54%); the sensitivity is 51.75% (48.35%, 55.13%); the specificity is 100% (99.77%, 100%). The Kappa coefficient is only 0.590 (0.555, 0.626) (Figure 2C), indicating that the consistency between ESPEN 2015 and SGA is moderate.

### 3.5. Adverse Clinical Outcomes of the Patients within 30 Days of Hospitalization 

According to the three diagnostic criteria, all patients were categorized into two subgroups, malnutrition and normal, and their adverse clinical outcomes within 30 days of hospitalization were compared between subgroups. Table 2 shows that the total number of subjects with complications was 103 (4.1%), including 62 infectious complications (2.5%) and 41 non-infectious complications (1.6%). 162 patients (6.4%) were transferred to ICU, and 10 patients (0.4%) died during hospitalization. No matter which criteria were adopted, the incidence of total complications and mortality and length of stay (LOS) in malnourished patients were higher than those in non-malnourished patients (*p* < 0.05). The incidences of infectious and noninfectious complications and the total hospitalization expenses of malnourished patients diagnosed with the GLIM criteria were higher than those of non-malnourished patients (*p* < 0.05). The incidence of infectious complications in malnourished patients diagnosed with SGA was higher than that in normal patients (*p* < 0.05). According to ESPEN2015, only the incidence of noninfectious complications in malnourished patients was higher than that in non-malnourished ones (*p* < 0.05). According to the three diagnostic criteria, there were no significant differences between malnourished and normal patients in terms of ICU admission and days in ICU (*p* > 0.05). Collectively, according to the GLIM criteria, the incidence of total complications, infectious complications, non-infectious complications, length of hospital stay, and total hospitalization expenses of malnourished people are significantly higher than those of non-malnourished people, which can predict more adverse hospitalization outcomes and is the most sensitive criteria to predict adverse hospitalization outcomes among the three diagnostic criteria for malnutrition, although the significance of the prediction needs to be verified by more analysis and more studies.

### 3.6. Covariates Analysis of the Exposure and Outcome Variables

Spearman’s rank correlation coefficient in Supplementary Table S1 shows that age, gender, and marital status are independent exposure variables of the subjects among all demographic characteristics, anthropometric parameters, hematological parameters, and reasons for hospitalization. Supplementary Table S2 shows that the total incidence of in-hospital complications is significantly and positively correlated with that of the infectious complications, and non-infectious complications, and so LOS is taken as the main outcome variable in the present study. Because Table 2 shows that the frequencies of mortality, ICU admission, and days in ICU are too small to be statistically analyzed, they are not used as outcome variables in the following statistical analysis.

### 3.7. Factors Influencing the Total In-Hospital Complications in the Patients

To investigate the factors affecting the total complications of elderly patients during hospitalization, we took it as the outcome variable and age, gender, marital status, along with the malnutrition diagnosis with the GLIM, SGA, and ESPEN2015 criteria, respectively, as independent exposure variables in the binary logistic regression analysis as model 1, model 2, and Model 3. As displayed in Table 3, the ORs of the GLIM, SGA, and ESPEN 2015 criteria are 2.414, 1.745, and 1.786, respectively, indicating that the malnutrition diagnosis made with the GLIM criteria is more closely associated with the incidence of the total in-hospital complications of the subjects in the present study.

### 3.8. The Predictive Value of the Malnutrition Diagnosis with the Three Criteria on the Total In-Hospital Complications of the Patients

**Figure 3:**
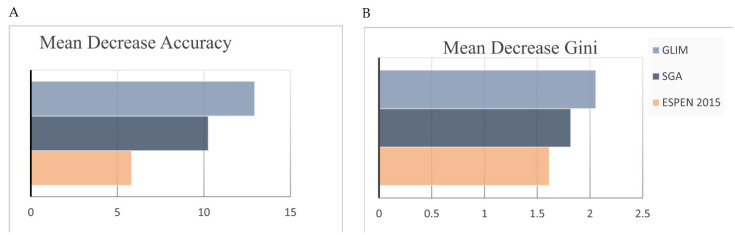
Mean decrease accuracy and mean decrease Gini coefficients of the three malnutrition diagnosis criteria predicted by the random forest algorithm. (**A**) Mean decrease accuracy. (**B**) Mean decrease Gini. GLIM, Global Leadership Initiative on Malnutrition; SGA, subjective global assessment; ESPEN 2015, the 2015 consensus statement by the European Society for Clinical Nutrition and Metabolism.

Figure 3 shows that the GLIM’s mean decrease accuracy (12.929) and mean decrease Gini (2.055) scores are higher than those of SGA (10.251 and 1.817, respectively) and ESPEN 2015 (5.819 and 1.614, respectively), suggesting that the GLIM criteria are more important than the other two criteria in predicting the incidence of the total in-hospital complications for the subjects in the present study.

## 4. Discussion

In the present study, the prevalence of malnutrition in the elderly inpatients diagnosed with the GLIM, SGA, and ESPEN 2015 criteria are 37.8%, 32.8%, and 17.0%, respectively. Malnutrition diagnosed by all three standards was associated with increased in-hospital complications, elongated hospital stays, increased medical expenses, and mortality. The results of traditional statistical methods and machine learning show that the GLIM criteria are superior to the other two criteria in predicting the total complications of the patients.

As a nation-wide multi-center study, the present study is useful for recognizing the prevalence of malnutrition in elderly patients in China. A previous multi-center study in elderly inpatients (average age 78.0 ± 5.7 years) in China revealed a malnutrition prevalence of 25.4–32.6% using the GLIM criteria. The proportion of tumor patients in this study is lower than the current study (14.9% vs. 37.5%) [27]. Tumor patients are more prone to malnutrition. Another study on Chinese patients reported that the detection rates of malnutrition according to the GLIM, PG-SGA, and ESPEN 2015 criteria in esophageal cancer resection patients (mean age 64.08 ± 7.74 years) were 33.3%, 23.1%, and 12.2%, respectively [18], consistent with the results of the present study. The age and ethnicity of the subjects are similar to the present study but the causes of being in hospital differ. In another study performed by Balci and Collogue [28], they found that the incidence of malnutrition defined by the SGA guideline was higher than the GLIM criteria (37.2% vs. 35.9%), which was inconsistent with the present study although the reasons for hospitalization are similar and the subjects were also recruited from the internal medicine and surgical wards. The reasons for the difference in the detection rate of malnutrition in the two studies may be related to several factors, such as the age and ethnicity of the subjects. The average age of the subjects in their study is 62 years, which is younger than that of the present study. The difference may also result from differences in other characteristics of the subjects and the specific implementation of the evaluators in their studies. After adjusting for the confounding factors, the authors believed that the GLIM criteria are superior to the SGA scale in predicting 5-year mortality. This is consistent with the current study that the GLIM criteria are superior to the SGA and the ESPEN 2015 criteria in predicting in-hospital complications.

There are many reasons for the increased complications in malnourished inpatients [29]. Malnutrition may increase catabolism, change body composition, induce oxidative stress and inflammation, reduce organ functions, and increase the incidence of various complications in hospitalized patients [29,30,31]. The impaired immune function of the malnourished patients will increase the chance of infection and progression of diseases, forming malicious cycles that further aggravate the nutritional status [32,33]. As a consequence, the efficacy of the treatment regimens may also be impaired in these patients [34].

In the present study, the performance of the GLIM criteria is superior to the SGA and the ESPEN 2015 criteria in predicting the in-hospital complications in elderly patients, consistent with previous studies. Using the random forest prediction, Yin, L et al. also found that the GLIM criteria are the best tool for predicting postoperative complications [18]. The SGA scale is a clinically verified tool for malnutrition diagnosis, especially in forecasting the hospitalization outcomes in tumor patients and surgical patients [11,35] but its accuracy in predicting the total postoperative complications of esophageal cancer patients is not ideal [18]. Moreover, the implementation of the SGA scale requires that the physician has systematic training in this regard. The evaluation procedure relies on the professionalism of the evaluator and inevitably has a subjective nature. The ESPEN 2015 framework attempted to make the malnutrition evaluation more objective than the SGA scale but failed to take into account the etiological factors causing malnutrition [2], resulting in its inferior performance to the other two criteria in the current study.

The operations in this study meet the requirements of the GLIM guidance on validation of the operational criteria for diagnosing malnutrition [3]. First, this study compared the GLIM criteria with the SGA scale, the semi-golden diagnostic criteria for malnutrition, and ESPEN 2015, when evaluating their powers as diagnostic criteria for malnutrition. Second, the effectiveness of the criteria in predicting complications was made by comparing to the SGA scale. Moreover, confounding factors are corrected when using the logistic regression and the random forest algorithm to elucidate the relationship between malnutrition defined by the three criteria and the total complications in hospitalized patients. Moreover, this study is based on a large-scale multicenter cohort study that was conducted around China and covers patients with a variety of diseases, so the results are representative.

This study has some limitations. First, this study is a retrospective study so we do not have data about reductions in muscle mass or the inflammatory indices. This situation is common in many retrospective studies. At present, the specific indicators for muscle mass reduction and inflammation, as well as their optimal thresholds, in the diagnosis of malnutrition are still unknown. Many researchers have adopted calf circumference plus grip strength as an alternative indicator of muscle mass reduction and serum albumin concentration reduction as an alternative indicator of inflammation [7,24]. This practice is also adopted in the present study. The use of anthropometric measures is supported by the GLIM guidance for the assessment of muscle mass reduction because it is easy to do in clinical settings where other methods for the assessment of muscle mass are not available [36]. This practice is also consistent with the original intention of using the GLIM as the minimum list of key indicators to identify malnutrition, which does not require expensive diagnostic equipment and can be applied in all parts of the world [3]. In future prospective studies we will explore the practicable indicators and optimal cut-off points of muscle mass reduction and inflammation and then use them to diagnose malnutrition more accurately, we will also verify the effectiveness of the diagnosis made by the GLIM criteria in predicting various clinical outcomes in a variety of patients.

## 5. Conclusions

The present study shows that the prevalence of malnutrition defined by the GLIM criteria is higher than that of the SGA and the ESPEN 2015 criteria in hospitalized elderly patients. Moreover, the GLIM criteria are superior in predicting in-hospital complications in elderly patients.

## Figures and Tables

**Table 1:** Comparison of the demographic characteristics, anthropometric parameters, blood parameters, and reasons for hospitalization between malnourished and non-malnourished subjects diagnosed with the GLIM, SGA, and ESPEN 2015 criteria, respectively.

Characteristics	GLIM			SGA			ESPEN 2015		
	Malnutrition	Non-Malnutrition	*p*	Malnutrition	Non-Malnutrition	*p*	Malnutrition	Non-Malnutrition	*p*
*n*	956	1570		829	1697		429	2097	
Age (year)	75.91 ± 7.28	73.85 ± 6.91	<0.0001	75.60 ± 7.15	74.16 ± 7.06	<0.0001	76.66 ± 7.21	74.22 ± 7.03	<0.0001
Males (%)	584(61.09)	911(58.03)	0.129	512(61.76)	983(57.93)	0.066	255(59.44)	1240(59.13)	0.906
Married (%)	929(97.18)	1448(90.23)	<0.0001	796(96.02)	1581(93.16)	0.004	413(96.27)	1964(93.66)	0.036
Primary school and lower	705(73.74)	1108(70.57)	0.097	595(71.77)	1218(71.77)	0.102	322(75.06)	1491(71.10)	0.250
High school	125(13.08)	206(13.12)		122(14.72)	209(12.32)		49(11.42)	282(13.45)	
Bachelor’s degree or above	126(13.18)	256(16.31)		112(13.51)	270(15.91)		58(13.52)	324(15.45)	
Height (cm)	163.97 ± 8.45	164.41 ± 8.14	0.194	163.98 ± 8.38	164.37 ± 8.21	0.270	163.76 ± 8.30	164.34 ± 8.26	0.183
Weight (kg)	56.37 ± 10.88	65.15 ± 10.47	<0.0001	57.71 ± 11.08	63.84 ± 11.09	<0.0001	48.90 ± 7.42	64.48 ± 10.27	<0.0001
BMI (kg/m^2^)	20.90 ± 3.37	24.06 ± 3.14	<0.0001	21.40 ± 3.50	23.58 ± 3.38	<0.0001	18.17 ± 1.89	23.82 ± 3.03	<0.0001
Grip strength (kg)	19.83 ± 9.17	23.93 ± 9.27	<0.0001	20.42 ± 8.99	23.34 ± 9.51	<0.0001	18.78 ± 8.81	23.11 ± 9.40	<0.0001
Mid-upper arm circumference (cm)	25.04 ± 3.97	27.39 ± 3.27	<0.0001	25.28 ± 4.21	27.08 ± 3.28	<0.0001	23.42 ± 3.05	27.11 ± 3.56	<0.0001
Calf circumference (cm)	30.69 ± 3.96	33.68 ± 3.93	<0.0001	31.14 ± 4.43	33.20 ± 3.92	<0.0001	29.38 ± 3.57	33.18 ± 4.03	<0.0001
Lymphocytes ^§^ (10^9^/L)	1.30(0.46)	1.66(0.58)	<0.0001	1.30(0.45)	1.60(0.58)	<0.0001	1.22(0.42)	1.57(0.55)	<0.0001
Hemoglobin (g/L)	115.42 ± 21.95	126.92 ± 18.05	<0.0001	116.53 ± 22.08	125.42 ± 18.88	<0.0001	112.98 ± 21.38	124.38 ± 19.69	<0.0001
Total protein (g/L)	63.60 ± 7.29	66.63 ± 6.43	<0.0001	64.01 ± 7.25	66.19 ± 6.65	<0.0001	63.13 ± 7.22	65.92 ± 6.78	<0.0001
Albumin (g/L)	35.80 ± 5.37	39.74 ± 4.53	<0.0001	36.22 ± 5.51	39.22 ± 4.78	<0.0001	35.75 ± 5.41	38.70 ± 5.05	<0.0001
Pre-albumin (g/L)	0.21 ± 0.09	0.24 ± 0.07	<0.0001	0.21 ± 0.09	0.24 ± 0.07	<0.0001	0.21 ± 0.09	0.23 ± 0.08	0.004
Triglyceride (mmol/L)	1.47 ± 1.30	2.16 ± 2.22	<0.0001	1.53 ± 1.41	2.07 ± 2.15	<0.0001	1.33 ± 1.14	2.02 ± 2.07	<0.0001
Total cholesterol (mmol/L)	3.94 ± 1.36	4.18 ± 1.51	0.001	3.91 ± 1.44	4.17 ± 1.47	0.001	3.88 ± 1.40	4.13 ± 1.47	0.001
Endocrine diseases	18(1.88)	45(2.87)	0.124	19(2.29)	44(2.59)	0.649	6(1.40)	57(2.72)	0.110
Nervous system diseases	72(7.53)	358(22.80)	<0.0001	72(8.69)	358(21.10)	<0.0001	37(8.62)	393(18.74)	<0.0001
Osteoarthropathy	30(3.14)	111(7.07)	<0.0001	11(1.33)	130(7.66)	<0.0001	10(2.33)	131(6.25)	0.001
Digestive diseases	189(19.77)	274(17.45)	0.144	187(22.56)	276(16.26)	<0.0001	73(17.02)	390(18.60)	0.440
Respiratory diseases	101(10.56)	122(7.77)	0.016	90(10.86)	133(7.84)	0.012	51(11.89)	172(8.20)	0.014
Cardiovascular diseases	31(3.24)	107(6.82)	<0.0001	33(3.98)	105(6.19)	0.022	20(4.66)	118(5.63)	0.423
Tumors	483(50.52)	465(29.62)	<0.0001	390(47.04)	558(32.88)	<0.0001	213(49.65)	735(35.05)	<0.0001
Kidney diseases	3(0.31)	7(0.45)	0.608	4(0.48)	6(0.35)	0.628	1(0.23)	9(0.43)	0.415

Notes: BMI, body mass index; GLIM, Global Leadership Initiative on Malnutrition; SGA, subjective global assessment; ESPEN 2015, the 2015 consensus statement by the European Society for Clinical Nutrition and Metabolism. ^§^ Continuous variables that do not conform to the normal distribution are expressed as the median and the quartile deviation (QD).

**Table 2:** Comparison of the adverse clinical outcomes between malnourished and non-malnourished patients diagnosed with the GLIM, SGA, and ESPEN 2015 criteria, respectively.

	GLIM			SGA			ESPEN 2015		
	Malnutrition	Normal	*p*	Malnutrition	Normal	*p*	Malnutrition	Normal	*p*
*n*	956	1570	-	829	1697	-	429	2097	-
Total complications	60(6.3)	43(2.7)	<0.0001	47(50.7)	56(3.3)	0.005	27(6.3)	76(3.6)	0.011
Infectious complications	37(3.9)	25(1.6)	<0.0001	29(3.5)	33(1.9)	0.018	14(3.3)	48(2.3)	0.233
Non-infectious complications	23(2.4)	18(1.1)	0.015	18(2.2)	23(1.4)	0.128	13(3.0)	28(1.3)	0.011
ICU admission	62(6.5)	104(6.6)	0.891	50(6.0)	116(6.8)	0.444	24(5.6)	142(6.8)	0.267
Mortality	10(1.0)	0(0.0)	<0.0001	7(0.8)	3(0.2)	0.012	5(1.2)	5(0.2)	0.005
LOS, days ^#^	15.01 ± 6.83	13.89 ± 6.01	<0.0001	14.89 ± 6.82	14.03 ± 6.20	0.001	15.00 ± 7.13	14.17 ± 6.18	0.014
Days in ICU ^#,^^§^	0.00(0.00)	0.00(0.00)	0.557	0.00(0.00)	0.00(0.00)	0.479	0.00(0.00)	0.00(0.00)	0.400
Total hospital expenses, USD ^#,^^§^	3265.59(2592.52)	3242.81 (2285.47)	0.036	3242.81(2496.35)	3242.81(2333.12)	0.348	3052.32(2166.82)	3242.81(2510.13)	0.378

^#^ T-test or Mann–Whitney U-test were used, and the rest were Chi-square tests. -, not applicable; ICU, intensive care unit; LOS, length of stay; USD, US Dollar; GLIM, Global Leadership Initiative on Malnutrition; SGA, subjective global assessment; ESPEN 2015, the 2015 consensus statement by the European Society for Clinical Nutrition and Metabolism. ^§^ Continuous variables that do not conform to the normal distribution are expressed as the median and the quartile deviation (QD).

**Table 3:** Logistic regression analysis of the risk factors associated with the incidence of the total in-hospital complications in the elderly patients and the contributions made by the malnutrition diagnosis made with the GLIM, SGA, and ESPEN 2015 criteria, respectively.

Risk Factors	Model 1(GLIM)			Model 2 (SGA)			Model 3 (ESPEN 2015)		
	OR	95%CI	*p*	OR	95%CI	*p*	OR	95%CI	*p*
Malnutrition	2.414	(1.605–3.630)	<0.0001	1.745	(1.169–2.604)	0.006	1.786	(1.130–2.824)	0.013
Age	0.998	(0.971–1.026)	0.911	1.003	(0.976–1.031)	0.827	1.002	(0.975–1.030)	0.881
Gender	1.446	(0.947–2.208)	0.088	1.454	(0.953–2.219)	0.082	1.482	(0.971–2.261)	0.068
Marriage status	0.697	(0.314–1.547)	0.375	0.802	(0.364–1.767)	0.584	0.827	(0.376–1.821)	0.638

Notes: OR, odds ratio; 95%CI, 95% confidence interval; GLIM, Global Leadership Initiative on Malnutrition; SGA, subjective global assessment; ESPEN 2015, the 2015 consensus statement by the European Society for Clinical Nutrition and Metabolism.

## Data Availability

The original data supporting the conclusion of this paper will be provided by the author without improper retention.

## Supplementary Materials

The following supporting information can be downloaded at: https://www.mdpi.com/article/10.3390/nu14153035/s1, Table S1: Spearman’s rank correlation between the exposure variables of the subjects; Table S2: Spearman’s rank correlation between the in-hospital outcomes of the subjects.
